# Structured interactions explain the absence of keystone species in synthetic microcosms

**DOI:** 10.1093/ismejo/wraf211

**Published:** 2025-09-22

**Authors:** Sivan Pearl Mizrahi, Hyunseok Lee, Akshit Goyal, Erik Owen, Jeff Gore

**Affiliations:** Physics of Living Systems, Department of Physics, Massachusetts Institute of Technology, 77 Massachusetts Avenue, Cambridge, MA 02139, United States; Institute of Biochemistry, Food Science and Nutrition, The Robert H. Smith Faculty of Agriculture, Food and Environment. The Hebrew University of Jerusalem. 229 Herzl Street, Rehovot 7610001, Israel; Physics of Living Systems, Department of Physics, Massachusetts Institute of Technology, 77 Massachusetts Avenue, Cambridge, MA 02139, United States; Physics of Living Systems, Department of Physics, Massachusetts Institute of Technology, 77 Massachusetts Avenue, Cambridge, MA 02139, United States; International Centre for Theoretical Sciences, Tata Institute of Fundamental Research, Bengaluru, Karnataka 560089, India; Physics of Living Systems, Department of Physics, Massachusetts Institute of Technology, 77 Massachusetts Avenue, Cambridge, MA 02139, United States; Physics of Living Systems, Department of Physics, Massachusetts Institute of Technology, 77 Massachusetts Avenue, Cambridge, MA 02139, United States

**Keywords:** systems ecology, microbial communities, keystone species, secondary extinctions, community interactions, Lotka–Volterra

## Abstract

In complex ecosystems, the loss of certain species can trigger a cascade of secondary extinctions and invasions. However, our understanding of the prevalence of these critical “keystone” species and the factors influencing their emergence remains limited. To address these questions, we experimentally assembled microcosms from 16 marine bacterial species and found that multiple extinctions and invasions were exceedingly rare upon removal of a species from the initial inoculation. This was true across eight different environments with either simple carbon sources (e.g. glucose) and more complex ones (e.g. glycogen). By employing a generalized Lotka–Volterra model, we could reproduce these results when interspecies interactions followed a hierarchical pattern, wherein species impacted strongly by one species were also more likely to experience strong impacts from others. Such a pattern naturally emerges due to observed variation in carrying capacities and growth rates. Furthermore, using both statistical inference and spent media experiments, we inferred interspecies interaction strengths and found them consistent with structured interactions. Our results suggest that the natural emergence of structured interactions may provide community resilience to extinctions.

## Introduction

A long-standing question in ecology is how an ecosystem reacts to the removal of a resident species. Whereas the extinction of one species may leave a minimal impact on the community structure, the extinction of another species may lead to drastic changes, including cascades of extinction and the entry of invasive species. In his pioneering work Robert Paine coined the term “keystone” species [1], describing the starfish predator *Pisaster ochraceus* whose removal from the ecosystem led to a dramatic decrease in biodiversity. Other early identified keystone species included sea otters, whose role as an apex predator regulated the density of sea urchins and maintained kelp forests [2]. Over the years there were many debates on the definition of keystone species [3], both in terms of its meaning and how to quantify it. One accepted definition that emerged considers a keystone species to be a species that exerts a substantial impact on a community or ecosystem, disproportionate to its abundance [4]. Though, it is not completely obvious what is considered a proportionate impact.

A plethora of theoretical studies suggest that keystone species should be prevalent in natural ecosystems [5–9]. These studies focus on different properties of the species-interaction network to predict “keystoneness”. For example, highly connected species are believed to be more likely to serve as keystone species. Understanding the prevalence of keystone species within a community is essential both for conservation efforts and for community engineering.

A growing body of work suggests microbial communities are also likely to include keystone species [10–13]. Microbial communities offer practical tools to study the resilience of a system to species removal. Whereas direct removal of a species from an established community is challenging, synthetic communities enable systematic removal of each species from the initial inoculum—advancing the study of community assembly. Current microbial studies are fueled by high-throughput sequencing studies of different microbiomes and their sampling through time and space. Such serial data were used to infer the interactions among species and predict keystone species from network properties [12]. Utilizing synthetic microbial communities to perform community “drop-out” experiments, several studies have explored the impact that species absence has on a community [14–17]. Previous studies have identified certain species whose “dropping-out” significantly impact community structure (though not focusing specifically on secondary extinctions or invasions*)* [14, 17, 18]*.* Another study examined a synthetic seven-species maize-root community and identified *Enterobacter cloacae* as a keystone species whose removal from the initial inoculation of the community results in five secondary extinctions and the dominance of the remaining species [16]. These studies exemplify the use of synthetic microbial communities as a practical tool to identify species with a strong impact on community structure. Despite the identification of particular communities with a keystone species, the prevalence of keystone species across different environments remains unclear.

Environmental factors such as resource availability are thought to be important in determining the community structure and interspecies interactions type and strength [18–20] and thus might play a role in dictating the frequency of keystone species [18]. The interactions between different species can change in different environments, such that changing the resources can cause biotic-interactions to shift [20]. Thus, certain species can function as a keystone in one environment but not in another [18, 21]. In contrast to classical ecological models where keystone species are often top predators occupying distinct trophic levels, in microbial communities the metabolic abilities of certain species could dictate their role as keystone species, for instance, when they are performing a “key function” such as being the only efficient degraders of a recalcitrant substrate [22]. Changing the resources available within an environment could therefore alter the identity and even the presence of keystone species.

Here, we examined how frequently species absence from the community leads to multiple secondary invasions and extinctions, relative to the full community, and how the frequency of such cascades changes in different environments. We comprehensively assessed the prevalence of keystone species across a range of resources in bacterial microcosms, utilizing a synthetic marine microbial species pool to systematically explore the impact of each species’ removal from the initial inoculum. We assembled communities from the same pool of 16 species in eight distinct conditions, resulting in communities with varying species richness. Despite theoretical predictions suggesting that keystone species should be common, we find a complete absence of keystone species across all eight environmental conditions. Modifying the generalized Lotka–Volterra (gLV) model to include a hierarchical structure of interspecies interactions, expected to naturally arise from carrying capacity variation, recapitulates the lack of keystone species. We provide experimental evidence that species interactions in our communities indeed have such a structure. Together, our findings indicate that keystone species may be less common than previously assumed, partly owing to the natural emergence of structured interspecies interactions.

## Materials and methods

### Species, media, and carbon sources

We have used 16 isolates from the PRiME project of the Simons Foundation [23, 24], detailed in Table S1.

Marine broth (Difco 2261) (MB)—used for streaking, plating, and growth of starter cultures—supports the growth of all 16 species used in this work.

All experiments were carried in minimal media (Amaranth *et al.* [25], see in Supplementary Methods), with carbon sources listed in Table S2.

### Ecological knock-outs experiments

Colonies from all 16 isolates grown on MB-agar plates were picked and placed into culture tubes with 3 ml MB, grown at room temperature with shaking at 300 rpm for two days. ODs were measured and volumes equal to 0.006 OD_600_ were taken from each isolate. 17 assemblies were prepared: one inoculation with all 16 isolates and 16 with each isolates removed. These were diluted 1:80 into the respective minimal media with the relevant carbon source. For each community and media combinations there were either triplicate or duplicate samples. The growth dilution cycles were carried in 1 ml deep 96-well plates (Eppendorf EP951032701) filled with 400 μl, sealed with membrane (Excel Scientific AeraSeal sterile). For each cycle the plates were grown at 25°C, shaking at 300 rpm for 48 hr. Using a multichannel pipettor (Viaflo 96, Integra Biosciences, Hudson, USA) plates were mixed and 10 μl were transferred to 390 μl of fresh matched media, mixed, sealed, and incubated again. Following transfer, 100 μl remaining from the previous cycle were taken to a 96 well cell culture plate (VWR 62406-081) and OD_600_ was measured using a Tecan infinite 200 pro multiplate reader. The seventh cycle was frozen at −80°C and later taken for deoxyribonucleic acid extraction and 16S ribosomal ribonucleic acid (rRNA) gene amplicon sequencing.

## Results

**Figure 1:**
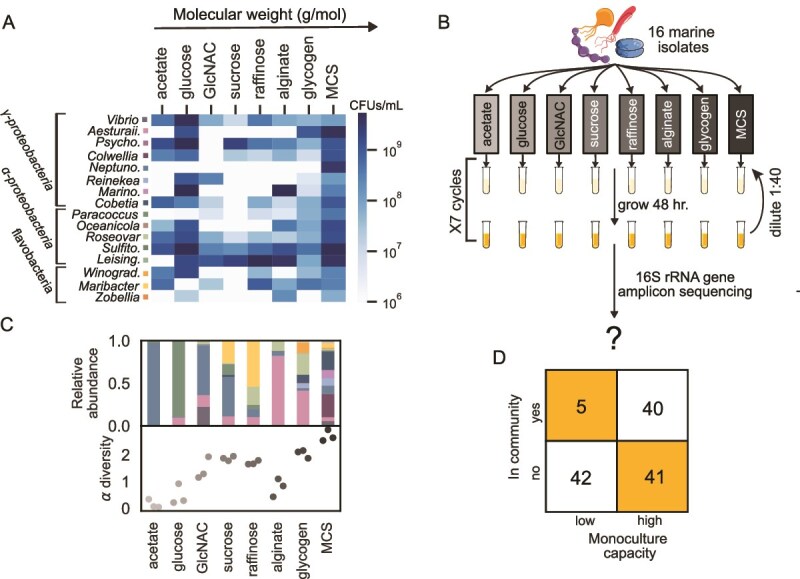
Synthetic microcosmos of 16 marine bacteria assembled on 8 different carbon sources differ in biodiversity and harbor multiple interactions. (A) Heatmap of the capacities (CFUs/ml) of each species’ monoculture at the end of four growth-dilution cycles. Each row designates a different species, ordered according to their family phylogenetic classification. (B) Experiment layout: 16 marine bacterial species are inoculated in samples with minimal media with different carbon sources and are grown in 7 growth-dilution cycles of 1:40 every 2 days. At the end of the seventh cycle the samples are processed and sent to 16S rRNA gene amplicon sequencing to probe the community structure. (C) Biodiversity of the different communities. Top panel: the mean relative abundance of each species in each community over triplicates. Relative abundance is calculated for each sample: $\frac{\# species\ ASVs}{\# sample\ ASVs}$. Colors of species are same as in A. Bottom panel: the alpha diversity of each sample, using the Shannon index. (D) Growth capability as monoculture is not necessarily indicative of survival in the community. Count table of all species capacities in monoculture (below or above 10^7^ CFUs/ml) and their appearance in the community (>0.004% relative abundance).

To study the sensitivity of a community to single species absence we need to consider both the community members and the environment, which plays a significant part in shaping the community structure. Species have different properties in different environments (e.g. different growth rates, carrying capacities), and interspecies interactions between species can also change between different environments. We chose to study a pool consisting of 16 bacterial species isolated from marine coastal waters that are representative of 5 major families (*Flavobacteriaceae*, *Rhodobacteraceae*, *Oceanospirillaceae*, *Alteromonadaceae*, and *Vibrionaceae)* that could be differentiated by 16S rRNA gene amplicon sequencing (Table S1). In addition, we studied eight different environments that differ from one another by their carbon source (Table S2). The carbon sources we used range in complexity from being very simple molecules (such as acetate and glucose) that are accessible to most species to long polymers (such as alginate and glycogen) that require specialized enzymes to break down. We also included an environment composed of many different carbon sources such as amino acids and sugar sources (denoted MCS, for multiple carbon sources, which is a dilution 1:5 of Marine Broth, see Supplementary methods). When grown by themselves in monoculture the species display large variability in growth capabilities, with less than half of the species displaying significant growth on sucrose but with all species growing well in the MCS media (Fig. 1A). The wide range of environments, together with the associated variation in species’ ability to grow on the provided carbon source, increases the likelihood that our assembled communities have a variety of different interactions and dependencies.

### Communities assembled on different carbon sources harbor multiple interactions

We assembled communities by inoculating media of the different environments with all 16 species, which we refer to as “full” communities. The full communities were grown in triplicate and diluted 1:40 every 2 days for 7 cycles (Fig. 1B). 16S rRNA gene amplicon sequencing revealed that the community composition of the full communities varied with environment, with greater biodiversity in the more complex carbon sources (either with higher molecular weight or being MCS with many different carbon sources). For example, whereas the acetate environment was inhabited by one dominant species (and one other species with very low frequency that crosses our threshold of 0.4% in only one of the triplicates), the MCS environment harbored 10 abundant species without distinct dominance (Fig. 1C). Many of the species that were unable to grow well in monoculture were nonetheless able to co-occur in the community (Fig. 1D). At the same time, many of the species that grew well in monocultures were unable to survive in the community (Fig. 1D). Both observations suggest that both beneficial and competitive interactions are widespread within our communities.

### Lack of keystone species in experimental marine communities

We developed an experimental framework to assess the likelihood of secondary extinctions and invasions of community members resulting from the removal of a particular species from the community. Because removing a specific species from the assembled community is technically challenging, we used an alternative approach we termed “Ecological Knock-Outs” (EKOs) in which we assembled distinct communities by systematically removing a specific species from the initial inoculum used during community assembly (sometimes called drop-out experiments). Species that were present in the full community but were not present in the assembled EKO community constituted secondary extinctions, whereas species that were not in the full community yet were in the EKO constituted secondary invasions. To determine which species are secondarily impacted and address noise in our dataset, we used thresholds and a majority-decision rule based on replicates. A species is considered impacted if the majority of replicates fall above or below multiple thresholds compared to the majority of replicates in the full community (see Materials and Methods). A keystone species would be a species whose EKO leads to multiple secondary impacts (SI; both invasions and extinctions).

To characterize the frequency of keystone species we experimentally performed knockouts of each of the sixteen species in each of the eight environments. We quantified the impact of each EKO on community structure using three complementary ways. First, following published work [17], we compared the observed EKO community to a predicted community generated by renormalizing the full community after setting the EKO species relative abundance to zero (we termed “EKO distance from Full”). Second, we identified secondary extinctions and invasions by detecting species whose presence or absence in the EKO community differed from that in the full community. Species present in the full community but absent from the assembled EKO community constituted secondary extinctions, whereas species absent from the full community yet present in the EKO community constituted secondary invasions. A keystone species would be a species whose EKO leads to multiple SI (both invasions and extinctions) (termed “secondary impacts”). Third, we used ANOVA followed by pairwise comparisons to detect species with significantly different abundances across communities and identify which EKOs caused these differences (termed “differential abundance”, see Supplementary Methods). Together, the first method quantifies overall community compositional change, whereas the others highlight specific species affected by each knockout.

**Figure 2:**
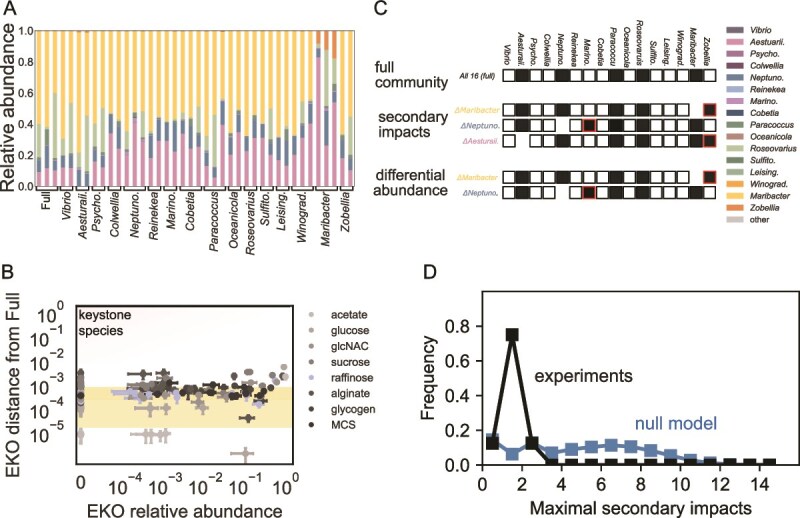
Lack of keystone species in experimental marine microcosms assembled in eight different carbon sources. (A) The relative abundances of all replicates of the full and EKO communities grown on raffinose. The species name below each bar group denotes the EKO of the replicates. (B) EKO distance from the full community across all EKOs on various carbon sources. Circles represent the mean of the computed distances between each EKO replicate and each prediction from full community replicates. The yellow shaded region indicates the “noise” range (lowest to highest) derived from all media, calculated as the mean distance among full community replicates. The red shaded zone highlights EKOs that are less abundant and exhibit larger distances from the full community, representing the most keystone-like species. (C) Schematic illustration of species survival of the full (top) and all EKO communities, communities in which an impact was identified either using SI (middle) or differential abundance (bottom). Each row represents a community, and each column represents a species. Filled and empty squares indicate survival and extinction, respectively. A red frame indicates a secondary impact where this species’ presence differ from that in the full community. (D) Histogram of the maximum SI per EKO of all EKOs in all eight environments. This is a measure that reflects the EKO with most SI for a given ecosystem (carbon source/simulation), which describe the “keystone-ness”. The null model simulation is in blue, and the experimental data is in black.

To illustrate how these three methods can be applied, we examined raffinose data where the full community comprised five co-occurring species (Figs 1C and 2A). EKOs of eleven species absent from the full community caused no changes in their respective EKO communities. Among the five present species, only *Maribacter* EKO increased EKO distance from the Full community beyond noise (Fig. 2B). Because *Maribacter* is the most abundant species, it is unlikely a keystone. Secondary extinction and invasion analysis identified three EKOs causing secondary invasions (Fig. 2C): *Neptuno*. EKO led to *Marino* invasion, and *Maribacter* and *Aesturaii*. EKOs each led to *Zobellia* invasions. Two of these invasions were identified by differential abundance analysis as well. Overall, the modest SI observed did not trigger cascades or major community shifts, indicating no keystone species in raffinose.

Across all eight environments, the overwhelming majority of ecological knockouts (115 out of 128) resulted in no SI, with species composition remaining unchanged from the full community. Among the remaining 13 EKOs, 12 led to a single secondary impact, 11 of which were invasions, corresponding to the replacement of the absent species by another. The only secondary extinction observed was in glycogen. The most impactful EKO belonged to *Neptuno*, the dominant species on acetate, whose EKO led to the invasion of two species (Supplementary Fig. S1). Given the potential for up to 15 SI, even *Neptuno* likely does not qualify as a keystone species in acetate by most definitions, especially as it is the most abundant species in the full community.

The limited impact of EKOs on other species was consistent across methods. We identified nine EKOs causing statistically significant changes in species abundance, seven of which overlapped with the secondary impact analysis. EKO distances from Full varied by environment, with sucrose and alginate showing larger shifts driven by fluctuations in low-abundance species (*Cobetia*, *Colwellia*) occurring not only between EKOs but also within them, and thus were not detected by differential abundance analysis. Some higher EKO distances from Full resulted from EKOs of highly abundant species (e.g. acetate, glucose, GlcNAC) or were replicate-specific (e.g. *Oceanicola* and *Roseovarius* in MCS), so these do not clearly indicate keystone species. Because no method detected keystone species (Fig. 2A–C, Supplementary Fig. S1), we focused on SI, which are intuitive and strongly overlap with differential abundance results.

Even though we rarely observed multiple SI resulting from a single EKO, in some cases community biomass and structure did indeed change in the EKOs of certain species (Supplementary Fig. S2). Moreover, although our study focused on quantifying keystone species abundance, our data also reveal mechanisms underlying community structure and function linked to the distinction between primary degraders and non-degraders of the supplied carbon source (see Supplementary Data). Experimental knockouts of the primary degrader *Aestuarii* led to invasions of other degraders, emphasizing complex interspecies interactions (Supplementary Fig. S3). Despite these observations, only one secondary impact was detected, indicating a limited effect. Overall, our ecological knockout experiments reveal a striking resilience of our communities to species absence, with a lack of keystone species.

### Interaction matrix with a hierarchical structure decreases the likelihood of secondary impacts

In our synthetic marine communities, we observed a low rate of SI over a wide range of environments. Is such, a lack of keystone species what we should expect from a generic ecological community? To address this, we implemented a generalized gLV model to simulate community assembly and EKOs of interacting species:


(1) \begin{equation*} {\dot{n}}_i={r}_i\left(1-{n}_i-{\sum_{i\ne j}}{\alpha}_{ij}{n}_j\right){n}_i \end{equation*}

**Figure 3:**
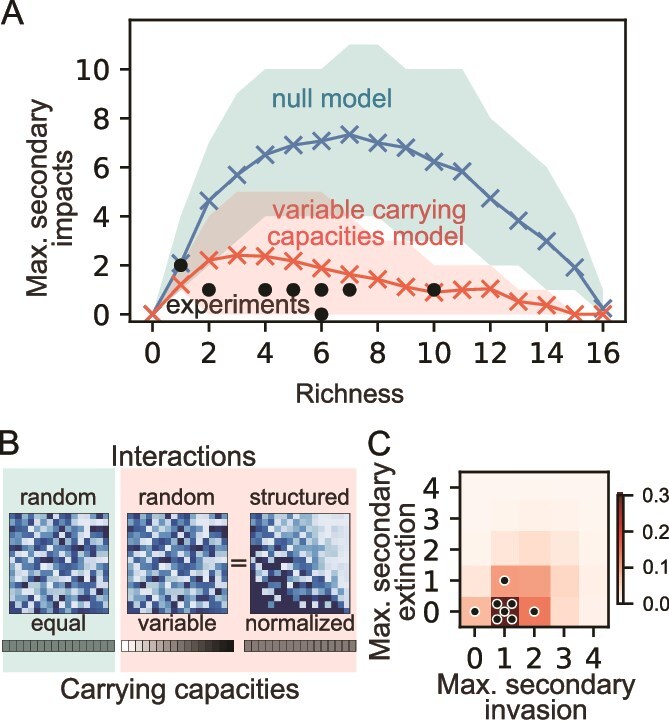
The gLV model can recapitulate the low number of SI when structured interactions due to variation in species carrying capacities are included. (A) Maximum SI for a given EKO in a simulation set/environment as a function of the richness of the full community. For the experimental data, each dot denotes the result of a certain environment (carbon source). For the simulations the mean over all simulations with a given full community richness appears in the plot, whereas the shaded regions denote the spread of the 10%–90% of the data of each richness. The null model simulation is in blue; structured correlations model simulation is in red, and the experimental data is in black. (B) Representative interaction matrices of the null model (left panel), in which the interactions are drawn independently and identically distributed from a certain distribution (a uniform distribution is shown here), or a model incorporating species’ carrying capacities into the interaction matrix (middle and right panels). The middle panel displays a model with interaction matrix drawn from uniform distribution and the introduction of variable carrying capacities, whereas the right panel displays an equal model in which the carrying capacities have been normalized and the interactions have been scaled accordingly (equation 3). Color denotes interaction strength with light blue equal to 0 and dark blue to 1.5. (C) Density plot of the frequency of simulations/experiments resulting in the stated number of maximum SI, separately considering extinctions and invasions. In red is the color relevant for the frequency of this result in structured correlations simulation set. Each black dot represents an experimental environment.

Here, ${n}_i$ is population size that is normalized to each species’ carrying capacity. ${r}_i$ is growth rate of species $i$, and ${\alpha}_{ij}$ is the interaction coefficient denoting impact from species $j$ to species $i$. As a starting point, we simulated community assembly from 16-species pools with random interactions ${\alpha}_{ij}\sim Uniform\left(\mu -\sigma, \mu +\sigma \right)$, as we sweep over interaction strength $\mu$ and variation $\sigma$ to represent a wide range of environment (Fig. 3A, Methods). This null model could capture variations in the number of co-occurring species in the full communities in different resource environments, as the community diversity decreased with increasing mean and variance of the interaction strength (see Discussion).

To investigate the SI in our model, we calculated three metrics for each simulated species pool: (i) the number of co-occurring species (richness) in the full community, (ii) the sum of all SI in each simulation species pool (total SI) and (iii) the maximum number of SI across all 16 EKOs (max SI). Our null model simulations showed a high number of max SI (4.4 ± 3.0), suggesting that we should naively expect most communities to have keystone species, as the absence of at least one species is expected to affect more than a quarter of the remaining species (Fig. 2D). However, in contrast to the null model, our experimental observations revealed no keystone species. Specifically, among the eight environments, six had a max SI of one, one environment had a max SI of two, and another had none.

Most simulation sets of our null model resulted in a high total number of SI (18 ± 15), with 25% of individual EKOs leading to two or more SI. In contrast, we experimentally observed an average of 1.8 ± 1.0 SI per environment (total SIs), and only 1 out of 128 of EKOs resulting in two SI. Our gLV null model therefore massively over-predicts the number of SI relative to what we observe experimentally.

What could account for the discrepancy between our null model, which predicted numerous keystone species, and our experimental results, which demonstrated strong resilience to species EKOs? One possibility is that our null model neglected variation in carrying capacities between species as we considered interactions between populations normalized to their own carrying capacities. Indeed, our experimental species pool exhibited a wide range of carrying capacities in every environment (Fig. 1A), which was not explicitly considered in our null model. Therefore, as our revised model, we used a gLV model with variation in carrying capacities:


(2) \begin{equation*} {\dot{N}}_i={R}_i\left({K}_i-{N}_i-{\sum_{i\ne j}}{A}_{ij}{N}_j\right){N}_i, \end{equation*}

where ${K}_i$ is the carrying capacity of species $i$. ${N}_i$ is now the population size without normalization, and ${R}_i$ and ${A}_{ij}$ are converted accordingly (see SI Appendix). As before, we considered random interactions ${A}_{ij}\sim Uniform\left(\mu -\sigma, \mu +\sigma \right)$, as we sweep over interaction strength $\mu$ and variation $\sigma$. Then we asked whether, unlike the null model case, this model with variation in ${K}_i$ can recapitulate the lack of keystone species.

We found that, as the variability of carrying capacities increased, SI became rarer in our simulations. In the case of a uniformly distributed carrying capacity between 0.1 and 1.9, we observed 1.9 $\pm$1.3 max SI. Similarly, we observed a low total number of SI (3 ± 3) and 95% of individual EKOs in simulations led to 0 or 1 SI (Fig. 3A). Inclusion of variation in species carrying capacity in our model therefore recapitulated the small number of SI that we observed experimentally. Both with and without carrying capacity variation we observe a non-monotonic connection between SI and diversity, with intermediate richness leading to higher likelihood for SI (Fig. 3A). In addition, measures of keystone species based on EKO distances from Full, instead of the number of SI, also recapitulate that the null model leads to keystone species whereas carrying capacity variation suppresses them (Supplementary Fig. S4).

How did the inclusion of carrying capacity variation reduce the SI? In the framework of gLV model, adding carrying capacities is equivalent to rescaling the interspecies interactions (Fig. 3B, Equation 3, Appendix):


(3) \begin{equation*} {\alpha}_{ij}=\frac{K_j}{K_i}{A}_{ij} \end{equation*}

This indicates that, in terms of the normalized effective interactions ${\alpha}_{ij}$, species with a large carrying capacity therefore exert a stronger impact on species with a small carrying capacity. Relatedly, a species with a large carrying capacity is likely to be less impacted by all other species, which is reflected in the structure of the effective interactions (Fig. 3B right panel, Appendix). Therefore, our simulations suggest that structured interactions may prevent the emergence of keystone species.

It is natural to expect structured interactions in nature for many reasons including variation in carrying capacity and variation in growth rates coupled with mortality or dilution. However, it is counterintuitive that such structure would prevent, not promote, keystone species, given that a species with large carrying capacity might be expected to have a profound effect on a community, and removing the species might therefore lead to a cascade of SI as the community re-orders itself. To better understand how the emergence of keystone species depends on different types of structures, we took a closer look into the structure induced by carrying capacity. Carrying capacities introduce correlations to the pairwise interactions in a bi-directional manner: species with higher carrying capacities are likely to both strongly affect other species and to be weakly affected by others. We dissected the two directional effects by simulating EKOs with correlated interactions based on either affecter ($\langle{\alpha}_{ik}{\alpha}_{jk}\rangle >0$) or affected ($\langle{\alpha}_{ki}{\alpha}_{kj}\rangle >0$) species:


(4) \begin{equation*} {\alpha}_{ij}={K}_j{A}_{ij}\ \left(\mathrm{affecter}\ \mathrm{based}\right),\kern0.5em {\alpha}_{ij}=\frac{1}{K_i}{A}_{ij}\ \left(\mathrm{affected}\ \mathrm{based}\right) \end{equation*}

We found that only the affected-based correlation in interspecies interactions reduced SI, whereas the affecter-based correlation did not change the statistics of SI (Supplementary Fig. S5). Perhaps in the presence of affected-based correlations, because a species is inhibited by a similar degree by all other species, removal of another species is unlikely to significantly change the overall inhibition on it. This shows that structured interactions from carrying capacities or affected-based correlations can make communities resilient to removal of a species.

In addition to predicting that SI are rare, the variable carrying capacity gLV model also predicts that secondary invasions will be more common than secondary extinctions (with our base sampling parameters, simulations yielded 2.6 $\pm$1.9 maximum secondary invasions across all 16 EKOs and 0.8 $\pm$1.6 maximum secondary extinctions). Consistent with this prediction across the eight environments we observed 1.0 $\pm$0.5 maximum secondary invasion across 16 EKOs and 0.1 $\pm$0.3 maximum secondary extinctions (Fig. 3C). This contrasts with the null model in which the number of secondary invasions and extinctions are not only larger but also more balanced (Supplementary Fig. S6).

### Inferred interspecies interactions display a hierarchical structure

We sought to test whether the data from our experimental communities were consistent with structured interactions, predicted to reduce the prevalence of keystone species in gLV models. We did this in two complementary ways: (i) using statistical inference to fit the interaction strengths and infer any evidence of structure, and (ii) using spent media experiments to analyze the impact of each species’ spent media on the growth of all other species.

**Figure 4:**
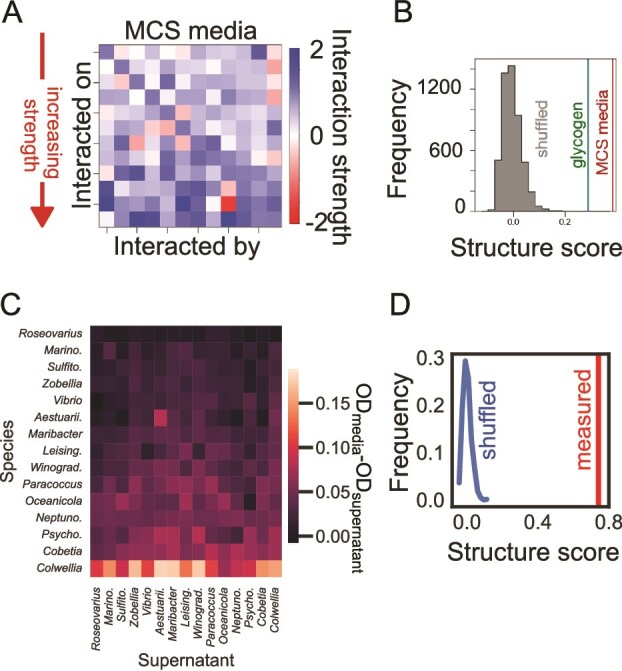
Inferred interaction matrix and spent media experiment suggest a hierarchical interaction structure between species. (A and B) (Left) inferred interspecies interaction matrix from EKO community data in MCS media using our statistical approach. Species are ordered by increasing average interaction strength along rows (A). Structure score quantifying the mean correlation between columns of an interaction matrix (methods) shown for matrices from MCS and glycogen media (B). Histogram shows a comparison with shuffled versions of these matrices. The matrices are much more structured than expected by chance. (C) Measured supernatants impacts, calculated by the difference of OD at the end of 2 days of incubation on the supernatant and that of the original media. The rows are ordered by the least impacted species (mean impact from all species supernatants) to the most impacted. Similarly, columns are ordered by the least impacting supernatants to the most impacting. *Reinekea* sample was contaminated and removed from the analysis. (D) Structure scores of the impacted species correlations. In red: the scores from the matrix appearing in C. In blue: a histogram of the scores of 1000 shuffles of the matrix entries*.*

We developed a statistical inference procedure to find the most likely matrix of interaction strengths that explained the observed species’ abundance data in different knockout experiments (Fig. 4A, see Methods). Briefly, our method used measurements of species’ individual carrying capacities (monoculture OD), relative abundances (from 16S rRNA gene amplicon sequencing) as well as total community biomass (community OD), and assuming a Lotka–Volterra model, aimed to infer all interspecies interaction strengths that best explained the data. Because the number of fit parameters (interaction strengths) was larger than the number of data points (knocked out species), we used an appropriate penalty to avoid overfitting and used bootstrapping to infer a family of interaction matrices. For each matrix, we quantified the extent to which it had the structure expected from theory: namely the mean correlation between its columns (see Methods). We found that the distribution of inferred matrices had a much larger mean correlation than randomly shuffled versions of them (Fig. 4B). This was true across matrices inferred using all media tested (Supplementary Fig. S7). Species abundances within communities were therefore consistent with the structured interactions as expected from theory.

As an independent way to infer metabolite-mediated interspecies interactions, we grew each species in the spent media of each other species. We quantified the relative growth of species *i* on the spent media of species *j,* compared with its growth in monoculture. This measure indicates the extent to which the environment created by species *j* promoted or hindered the growth of species *i*. We focused on the MCS media because all species were able to grow on it as monocultures (Fig. 1A), as well as it being the media with the highest richness of species (Fig. 1C). We found that the impacts a species experiences from other species supernatants were correlated and vice versa (Fig. 4C). This correlation was far higher than any correlation calculated on the shuffled matrix (Fig. 4D, Supplementary Fig. S8 displays reproducibility), exemplifying the statistical significance of the correlation in the inferred interactions. We also observed similar correlations within a different species pool and environments by re-analyzing published data on pairwise interactions (Kehe *et al.* [26], Supplementary Fig. S9). Our spent media experiments therefore support the presence of structured interactions within the community, which provide a natural explanation for the absence of keystone species observed in our experiments.

## Discussion

In this work we used synthetic microcosms to systematically measure the frequency of secondary extinctions and invasions following the removal of each of the constituent species from initial inoculation. This approach is different from removing species from established communities, which is experimentally challenging. However, we expect that a keystone species—whose removal from an established community would have a strong impact—would also impact community assembly, when excluded from the initial inoculum. To understand how SI depend on environmental resource complexity, we conducted our experiments using the same set of 16 species in eight distinct environments across a range of carbon sources with varying complexity. Although sampling occurred only after seven cycles and stable states cannot be guaranteed, replicate similarity suggests relative stability. However, some species fluctuated independently of EKOs, likely due to stochastic factors. Thus, we interpret species roles from EKO-driven compositional changes, acknowledging potential future community shifts. The assembled communities had varying levels of richness, ranging from 1 to 12 co-occurring species.

Whereas definitions of keystone species vary and depend on context, our approach employs three complementary methods to capture them. Strikingly, none of the methods was able to identify any keystone species in all eight environments. Focusing in the conservative SI method this finding made even more striking through comparison with a null model based on the gLV framework, which predicts that multiple SI should be common. This lack of SI was observed even in EKOs of species that are abundant within the original community, highlighting that our definition of keystone species is even less stringent than commonly used (where the impact of species EKO is large compared with the abundance). Thus, we concluded that there were no keystone species in our microcosms in any environment.

In this work, we leveraged synthetic communities to focus on shifts in community structure without delving into the mechanistic drivers of community dynamics. Our findings, such as the effect of EKO of primary degraders of alginate and glycogen on community composition (Supplementary Fig. S3), suggest that metabolic capabilities are important in community assembly. Given the complexity and functional redundancy typical of natural microbial communities, future studies integrating metabolic and functional analyses will be crucial to uncover the mechanistic basis of microbial interactions and to better identify keystone species.

We found that introducing correlations between the rows of the interaction matrix reduces the incidence of SI and thereby greatly diminishes the prevalence of keystone species in communities. This was also true in decreasing the number of EKOs with low relative abundance leading to large EKO distances from Full (Supplementary Fig. S4). The correlations that emerge in our model with carrying capacity variations lead to a hierarchical structure of species interactions. Such correlations naturally arise when we consider the experimentally observed variation in carrying capacities or growth rates and mortality rates across different species in the gLV equations (Supplementary Fig. S10). Variation in any or all these quantities is expected, and results in an effective interspecies interaction matrix which is correlated or hierarchically structured (Fig. 3B), even though the underlying species-species interactions may be unstructured. The model also predicts that communities with intermediate richness tend to show the greatest number of SI (Fig. 3C, blue line), a pattern that can be understood via a simple theoretical argument based on random re-sampling of surviving species (Supplementary Discussion). However, SI in our experiment are too rare to confirm that such a pattern appears. By statistically inferring the effective interaction matrices using our community data, we indeed found that they possess such a hierarchical structure as expected from theory (Fig. 4). Additionally, we measured pairwise interactions in spent media experiments. Although this approach primarily captures secreted metabolite effects and may miss contact-dependent interactions, low-concentration signaling molecules, or growth-phase-specific compounds, we observed a similar hierarchical structure. An analysis of thousands of pairwise interactions measured in a droplet-based microfluidic chip [26] show that these communities display similar structure as well (Supplementary Fig. S9). Our results contribute to a recently growing understanding that soil and plant microbial communities might have a competitive hierarchy [27, 28]. Overall, we believe that such structured interactions are abundant in microbial ecosystems and can provide resilience in the face of species extinctions and invasions.

Our results stand in contrast with several recent studies which performed similar EKO experiments to study microbiomes associated with specific environments such as plants [14–16] and a “synthetic gut” [17, 18]. These studies aimed to identify species with “keystone” properties, using various metrics such as their influence on the host and changes in community structure. However, even when we relaxed our requirement for secondary invasions or extinctions and examined differences in species’ relative abundances between the full and EKO communities, our results generally support the lack of keystone species. Only one EKO showed significant changes in abundance in three species compared to the full community (Supplementary Fig. S1B). When focusing on SI, two of these studies [17, 18] found a maximum of two SI, consistent with our findings. A third study [16] identified an EKO with five SI, possibly due to their meticulous selection of the species pool. Future studies should explore how the degree and incidence of keystone species depend on the underlying species pool, the environment, and the degree of ecological and evolutionary adaptation of the species to the environment.

To summarize, our exploration of species EKOs across multiple environments shows that extinction/invasion cascades are rare compared to naïve theoretical expectations assuming uncorrelated interspecies interactions. Incorporating correlated interspecies interaction structure into account can help future theoretical studies reflect common ecological patterns, aid in the identification of keystone species for conservation efforts and enable the design of stable microbial communities in industry.

## Supplementary Material

SupplementaryData_wraf211

## Data Availability

All amplicon sequencing data generated in this study can be accessed upon publication on the US National Center for Biotechnology Information SRA database under BioProject PRJNA1320718.
