# Supplementary material for: Structured interactions explain the absence of keystone species in synthetic microcosms
Source: ISME J. 2025 Sep 22;19(1):wraf211. doi: 10.1093/ismejo/wraf211 (PMC12510465; doi:10.1093/ismejo/wraf211)
Supplement: SupplementaryData_wraf211 [file supplementarydata_wraf211.pdf]

Structured interactions explain the absence of keystone species in synthetic microcosms

Sivan Pearl Mizrahi[1,2*], Hyunseok Lee[1], Akshit Goyal[1,3], Erik Owen[1], Jeff Gore[1*]

[1]Physics of Living Systems, Department of Physics, Massachusetts Institute of Technology, Cambridge 02139, USA.

[2]Institute of Biochemistry, Food Science and Nutrition. The Robert H. Smith Faculty of Agriculture, Food and Environment. The Hebrew University of Jerusalem. 229 Herzl Street, Rehovot 7610001, Israel.

[3]International Centre for Theoretical Sciences, Tata Institute of Fundamental Research, Bengaluru 560089, India

*Corresponding author

Conversion between gLV equations with and without carrying capacity variations

In the main text, we discuss two ways of writing generalized Lokta-Volterra models (eq. 1, eq. 2). Here, we will explicitly calculate the conversion from Equation 2, which models gLV dynamics of populations with varying carrying capacities, to Equation 1, which models gLV dynamics of normalized populations with respect to each species' carrying capacities.

Let us start from Equation 2:

$$\dot{N}_i = R_i\left(K_i - N_i - \sum_{i\neq j} A_{ij}N_j\right)N_i.$$

We now replace the population sizes N_i with normalized population sizes $n_i = N_i/K_i$:

$$K_i\dot{n}_i = R_i\left(K_i - K_i n_i - \sum_{i\neq j} A_{ij}K_j n_j\right)K_i n_i,$$

which can be simplified to

$$\dot{n}_i = R_i K_i\left(1 - n_i - \sum_{i\neq j}\frac{K_j}{K_i}A_{ij}\,n_j\right)n_i.$$

This is equivalent to Equation 1 with $r_i = R_i K_i$ and $\alpha_{ij} = \frac{K_j}{K_i} A_{ij}$.

Calculation of secondary impacts in the case of random assembly

In the main text, we discuss how the number of secondary impacts in simple Lotka-Volterra model reaches a null model prediction, i.e., the case of random assembly. Here we discuss how the number of secondary impacts can be calculated for the case of random assembly.

For the case of random assembly, we assume that the community assembly after an ecological knockout becomes completely independent of the community before the knockout, except that the average probability for survival over all species remains the same. In other words, this is a situation in which the survival of each species before the knockout does not provide any information on whether it can survive after the knockout. Under such assumption, we can calculate the expected number of secondary impacts in the following way.

Let us consider a situation in which the species pool size is S_0 and the chance of survival of a species in the given environment is S/S_0. In a specific scenario we expect to have S species survive in the full community. Now let us calculate the expected number of secondary impacts by considering two different situations.

First, let us consider the case in which a EKO was originally surviving, which has a chance of S/S_0. Then there are two ways that secondary impacts can occur: an originally survived species going extinct, and an originally extinct species surviving. In the former case, $S-1$ other species which originally survived, there is $1 - S/S_0$ chance that they go extinct. Similarly in the latter case, for $S_0 - S$ species which originally went extinct, there is S/S_0 chance that they survive. In the end, in the case in which an EKO species was originally surviving, the expected number of secondary impacts is $\frac{S}{S_0}\left(1 - \frac{S}{S_0}\right)\left(\frac{2S-1}{S_0}\right)$.

Next, let us consider the case in which a KO'ed species was originally going extinct, which has a chance of $(1 - \frac{S}{S_0})$. Then for S species which originally survived, there is $1 - S/S_0$ chance that they go extinct. Similarly, for $S_0 - S - 1$ species which originally went extinct, there is S/S_0

chance that they survive. In the end, in the case in which a KO'ed species was originally extinct, the expected number of secondary impacts is $\frac{S}{S_0}\left(1-\frac{S}{S_0}\right)\left(\frac{2S_0-2S-1}{S_0}\right)$.

Overall, the expected number of secondary impacts in the null model of random assembly is given by

$$2\frac{S}{S_0}\left(1-\frac{S}{S_0}\right)(1-\frac{1}{S_0})$$

<u>Metabolic link between community shift following EKOs</u>

In monocultures, only species possessing the enzymatic pathway to degrade alginate or glycogen (according to BioCyc[1]), hereafter termed 'degraders', reached carrying capacities above $3\text{x}10^8$ cells/mL. However, within communities, only *Aestuarii.*- a degrader of both alginate and glycogen—dominates, while other degraders are present at much lower relative abundances (less than 0.1% in alginate and 5.5% in glycogen, respectively, Supplementary Figure 3). Interestingly, non-degraders, despite their limited growth in monoculture, persist at substantial frequencies within communities (over 11% and 24% in alginate- and glycogen-grown communities, respectively). EKOs of *Aestuarii.* led to striking shifts: on alginate, its absence led to the invasion of *Maribacter*, another alginate degrader, while on glycogen, *Aestuarii.* EKO resulted in a marked increase in *Reinekea*, a glycogen degrader (this EKO has only a single replicate in glycogen thus it could not be identified by other methods as a significant impactful species in glycogen). Interestingly, *Reinekea* EKO on glycogen corresponds to the sole extinction in our dataset (of *Cobetia)*, suggesting a unique cross-feeding interaction might exist between *Reinekea* and *Cobetia*. Overall, our findings highlight that interactions go beyond a simple degrader–cross-feeder framework, likely involving complex feedbacks, competition, and metabolic dependencies, as suggested by prior work the role of secondary consumers in shaping community assembly on complex carbon sources[2–5].

Supplementary methods

Minimal media

Seawater (342.25 mM NaCl, 14.75 mM $MgCl_2$-$6H_2O$,1 mM $CaCl_2$-$2H_2O$, 6.75 KCl) mixed with trace minerals and vitamins (2.1 mg/L $FeSO_4$ * $7H_2O$, 0.03 mg/L H_3BO_3, 0.1 mg/L$MnCl_2$ * $4H_2O$, 0.19 mg/L $CoCl_2$ * $6H_2O$, 0.24 mg/L $NiCl_2$ * $6H_2O$, 0.2 mg/L $CuCl_2$* $2H_2O$, 0.144 mg/L $ZnSO_4$ * $7H_2O$,0.036 mg/L Na_2MoO_4 * $2H_2O$, 0.025 mg/L $NaVO_3$, 0.025 mg/L $NaWO_4$ $2H_2O$, 0.006 mg/L Na_2SeO_3 $5H_2O$, 0.1 mg/L Riboflavin, 0.03mg/L D-Biotin, 0.1 mg/L Thiamine hydrochloride, 0.1 mg/L L-ascorbic acid, 0.1 mg/L Ca-d- pantothenate, 0.1 mg/L Folate, 0.1 mg/L Nicotinate, 0.1 mg/L 4-aminobenzoic acid, 0.1 mg/L Pyridoxine HCl, 0.1 mg/L Lipoic acid, 0.1 mg/L NAD, 0.1 mg/L Thiamin pyrophosphate, 0.01 mg/L) Cyanocobalamin that were donated by the Cordero lab. HEPES 0.05 M, Nitrogen source: 0.01 M ammonium chloride, phosphorus source: 1 mM phosphate dibasic, sulfur source: 1 mM sodium sulfate. Different carbon sources were added as a function of the experiment. The carbon sources used are listed in Table S2 below, as well as their MW if applicable.

Table S1 - Strains

Isolate	Genus	Family	order	class
I2R16	*Psychosphaera*	*Pseudoalteromonadaceae*	*Alteromonadales*	*Gammaproteobacteria*
3B05	*Neptunomonas*	*Oceanospirillaceae*	*Oceanospirillale*	*Gammaproteobacteria*
G2M07	*Reinekea*	*Oceanospirillaceae*	*Oceanospirillale*	*Gammaproteobacteria*
C2R09	*Paracoccus*	*Rhodobacteraceae*	*Rhodobacterales*	*Alphaproteobacteria*
C3M06	*Oceanicola*	*Rhodobacteraceae*	*Rhodobacterales*	*Alphaproteobacteria*
E3R09	*Winogradskyella*	*Flavobacteriaceae*	*Flavobacteriales*	*Flavobacteriia*
E3R11	*Vibrio*	*Vibrionaceae*	*Vibrionales*	*Gammaproteobacteria*
A2M03	*Flavobacteriaceae*	*Flavobacteriaceae*	*Flavobacteriales*	*Flavobacteriia*
C3M10	*Leisingera*	*Rhodobacteraceae*	*Rhodobacterales*	*Alphaproteobacteria*
B3M08	*Sulfitobacter*	*Rhodobacteraceae*	*Rhodobacterales*	*Alphaproteobacteria*
C2M11	*Colwellia*	*Colwelliaceae*	*Alteromonadales*	*Gammaproteobacteria*
B2M13	*Cobetia*	*Halomonadaceae*	*Oceanospirillale*	*Gammaproteobacteria*

A3R04	*Aestuariibacter*	*Alteromonadaceae*	*Alteromonadales*	*Gammaproteobacteria*
C3R19	*Maribacter*	*Flavobacteriaceae*	*Flavobacteriales*	*Flavobacteriia*
D2R04	*Roseovarius*	*Rhodobacteraceae*	*Rhodobacterales*	*Alphaproteobacteria*
F3R08	*Marinobacter*	*Alteromonadaceae*	*Alteromonadales*	*Gammaproteobacteria*

Table S2 Carbon sources

Carbon Source	Final M	Final carbon M	Final %	MW (g/mole)
sodium acetate (Sigma 791741)	0.06	0.12	0.49	82.03
Glucose (Sigma G8270)	0.02	0.12	0.36	180.16
GlcNaC (Sigma A4106)	0.015	0.12	0.33	221.21
Sucrose (Macron 279110)	0.01	0.12	0.34	342.3
D-Raffinose pentahydrate (Amresco J392)	0.0067	0.12	0.4	594.51
sodium alginate (Sigma W201502)	0.0058	0.035	0.125	12000-14000
glycogen (Sigma G8751)	0.0019	0.045	0.125	270-3.5x10^6 (Personal correspondence with Sigma representative.)
MB diluted 1:5 (BD difco 279110)	NA	NA	0.0748	NA

All components mentioned here were weighted, mixed (titrated and stir-heated if needed) and filtered through a 0.2 µM PES filter.

DNA extraction and 16S-amplicon sequencing

Samples were defrosted, 200 µl were moved to 96 U-bottom well plates (Corning #353077), and centrifuged at 3,220 rcf for 3 minutes to pellet cells. Cells were washed with sterile water, pelleted again, and then supernatants were aspirated. Cells were resuspended in 25 µl of TES buffer (10 mM Tris-HCl, 1 mM EDTA, 100 mM NaCl). To lyse the cells, 250 U/µl of ReadyLyse (Lucigen R1804M) were added to the samples, followed by overnight shaking at room temperature. Samples were then centrifuged for 5 minutes at 3,220 rcf and supernatant was stored at -20 °C till shipment to sequencing. Prior to freezing, 1µl of each sample were taken to measure DNA concentration using Quant-it PicoGreen (ThermoFisher, P7589). Samples were sent to Aragonne National Laboratory where 16S-rRNA gene amplicon libraries were prepared with the 515F-860R primer set and sequenced on an Illumina MiSeq machine with a 2x151bp run.

Amplicon sequence variants (ASVs) were obtained using the R package DADA2. Filtering and trimming carried with the parameters: truncLen=c(150,150), trimLeft = 10, maxN=0, maxEE=c(2,2) and truncQ=2.

Taxonomic identities were assigned to ASVs using the SILVA version 138 database train set. R4.1.3 was used.

To match the assigned ASVs to any of the 16 species used in the experiment, BLAST (blastn version 2.13.0+) was run locally against a database containing the 16S sequences obtained from the 16 species. An ASV was assigned if there were no more than two mismatches between the query and the hit from the database. ASVs without a match were grouped as "other". 15 samples with 'other' assigned to more than 10% of the reads were removed from the analysis. Also 1 sample with less than 1000 reads was also removed from the analysis.

This resulted in a dataset of 312 samples, with a mean of 23,361 ± 10,147 reads per sample with an average frequency of less than 0.77% ± 1.62% of reads assigned as 'other'.

Inferring EKO impacts

We applied three approaches to assess whether the EKO of species i affects species j.

<u>Secondary impacts (threshold based)</u>

For a given threshold t, species i is considered occurring in a sample if its frequency exceeds t. Each community consists of replicates grown in the same media. We denote $O^{i,m}$ to be the mean of occurrences of species i in condition m over all replicates. Species i is considered present in condition m if it occurs in the majority of replicates for that condition ($O^{i,m}>0.5$).

Species i is considered invading in condition m if the difference in species i's occurrences between the EKO and the full community exceeds 0.5: $O^{i,m} - O^{i,full} > 0.5$ and extinct if its<0.5.

We have found a subset of species display high variability in their occurrences across all samples of a certain media. Species for which the mean occurrences between all samples in the given media (all EKOs and the full community) ranged between 0.2-0.8 considered inconsistent. We are assuming that in most EKO each species i is likely to have similar frequency as in the full community, while only in certain EKOs of impactful species it would differ. Large variability is unexpected and indeed was found to be unrelated to the background community in most cases. as their occurrences general seemed to be independent of the background community. For such species, we refined our analysis by comparing their mean frequencies in EKO communities to their overall mean frequency across all samples, considering only species whose mean frequency +/- std in the EKO are lower than the mean frequency -\+std over all samples as extinct\invading, respectively.

We have run this analysis on 13 thresholds: [0.001, 0.0015,0.002…0.007] and species were considered to experience secondary impacts if they were impacted in more than 6 of these thresholds.

This method tries to identify invasions and extinctions and thus ignores many differential abundances scenarios. Due to our relatively small synthetic communities' setup and small number of samples for each condition (<=3), many of the assumptions made in microbiome analysis frameworks that are used for differential abundance analysis are not suitable.

<u>Differential relative abundance (ANOVA based)</u>

We also conducted ANOVA on centered log-ratio transformed frequencies to detect species that exhibited significantly different means in specific EKO communities compared to others (a pseudocount of 5 was added for all data to avoid low count). While we acknowledge that the normality assumption of ANOVA may not be met, non-parametric Kruskal-Wallis tests did not

detect significant impacts, so we present the ANOVA results instead**.** Then for the significantly impacted species (p-value <0.05) we have searched for all significant EKOs that are significantly different from the full community, and at least three more communities (paired t-tests). This was done using the statsmodels module in python.

Difference in assembly

Difference in community assembly was computed as $\frac{1}{15}\sum_{i=1}^{15}\sum_{j=3}^{3}\sum_{k=1}^{r_x}\left(\hat{v}_{i,j,FULL} - v_{i,k}, x\right)^2$, where i represents species, X represents the EKO community lacking species X. $\hat{v}$ denotes the renormalized relative abundance of the full community when species X is removed from it. Here, $\hat{v} = \frac{v_i}{\sum_{l \in C} v_l}$ where C denotes the set of 15 inoculated species EKO community. j and r_x are the replicates of the renormalized full community and EKO community, respectively. Each dot represents the mean and SE of all j*r_x pairs.

Model and simulation

For carrying capacity, we use unit $K_i = (1, \dots., 1)$ for the null model and an arithmetic sequence $K_i = (0.1, 0.22, \dots, 1.78, 1.9)$ for the carrying capacity model.

For interspecies interaction, we sweep over two parameters (A_{min}, A_{max}) that determine the strength and variation of interaction matrix A_{ij} with steps size of 0.075. In other words, the distributions we use for sampling A_{ij} are $Uniform(0,0)$, $Uniform(0, 0.075)$, $Uniform(0.075, 0.075)$, …, $Uniform(1.5,1.5)$. For each parameter set (A_{min}, A_{max}), we draw 100 communities A_{ij} with 16 species. In this way, we sample total of 19000 communities for each model.

For each community (i.e. for each choice of A_{ij}), we numerically solve dynamics with initial conditions $N^{full}(t = 0) = (\frac{1}{16}, \dots, \frac{1}{16})$ for the full community simulation and $N^{EKO_i}(t = 0) = 0\ for\ species\ i,\ \frac{1}{16}\ for\ all\ others$ for 16 EKO simulations. We use numerical solver Tsit5 with automatic switching based on Rosenbrock method. This is done with package Differential Equations in Julia (v 1.9). Any species with population fraction < 0.01% at time $t = 1000$ after the onset of simulation is considered extinct.

Statistically inferring interactions from data

To infer the interaction matrices from the community abundance data, we took a maximum likelihood approach. Our method is inspired by recent approaches to infer interactions but operates in a different regime, one where coexistence is not common and ecological knock-out experiments do not provide enough data for unambiguous inference[6, 7]. For each species, we had measurements of their carrying capacities *K* from monoculture experiments, as well as relative abundances in knockout communities. For each knockout community, we also had measurements of the community biomass in the form of its total optical density (OD), which we used to convert each species' relative abundance to absolute abundance in units of OD. For each species gamma, we thus had a measured abundance N_obs, as well as a predicted abundance N_pred under a generalized Lotka-Volterra model (gLV) with an interaction matrix A to be determined. Generally, for any community with a set of surviving species (indicated by an asterisk *), the gLV prediction for the abundance of species gamma was given by:

$$N_{obs,\gamma} = \sum_{\delta=1}^{S} A_{\gamma\delta}^{-1} K_{\delta}$$

Assuming log-normally distributed errors (a reasonable assumption for microbiome abundance data[8]), we thus minimized the following cost function, which included the following two elements: (1) the total likelihood of observing a given set of species abundances in communities, as well as (2) a Ridge regularization penalty to avoid overfitting and ensure that the inferred matrices did not have arbitrarily large interaction strengths:

$$A_{opt} = \arg\min_{A}[\sum_{i=1}^{N}\sum_{\gamma=1}^{S}\left(\log N_{obs,\gamma} - \log N_{pred,\gamma}\right)^2 - \lambda\left|\left|A\right|\right|^2]$$

We used the matrix A_{opt} that minimized the above cost function as the inferred matrix for the given community data. Note that here, we used data from all knockout communities (represented by the

letter *i*) for a given growth medium and over all species present in that community (represented by the symbol γ), since we would generally expect different media to yield different interaction matrices for different communities.

Growth abilities on different species supernatants

Starter cultures were prepared twice, once for the supernatants and once for the species, in the following way: colonies from all 16 isolates streaked from frozen stock on MB-agar plates were picked, placed into culture tubes with 3 mL MB, and grown at room temperature with shaking at 300 rpm for two days.

For the supernatant cultures: each culture was diluted 1:40 in fresh MCS media for a total of 22.5 mL in 50 mL Falcon tubes. These cultures were grown at room temperature for 2 days, shaking. All cultures but that of *Reinekea* were filtered through 0.2 µm filter (ThermoFisher VWR#82030-938). *Reinekea* was filtered through 0.1 µm filter (ThermoFisher #565-0010), since this species can pass the 0.2 µm filter. These supernatants were diluted 1:1 with fresh MCS media.

For the growth experiment starter cultures from each species were diluted 1:40 in 400 µl of either supernatant or MCS media in triplicates. This was done in 1 mL deep 96-well plates (Eppendorf # 951033006). These plates were shaken for 2 days at RT. Then 100 µl of each sample, as well as background media were taken to have OD_{600} measured in a multiplate reader.

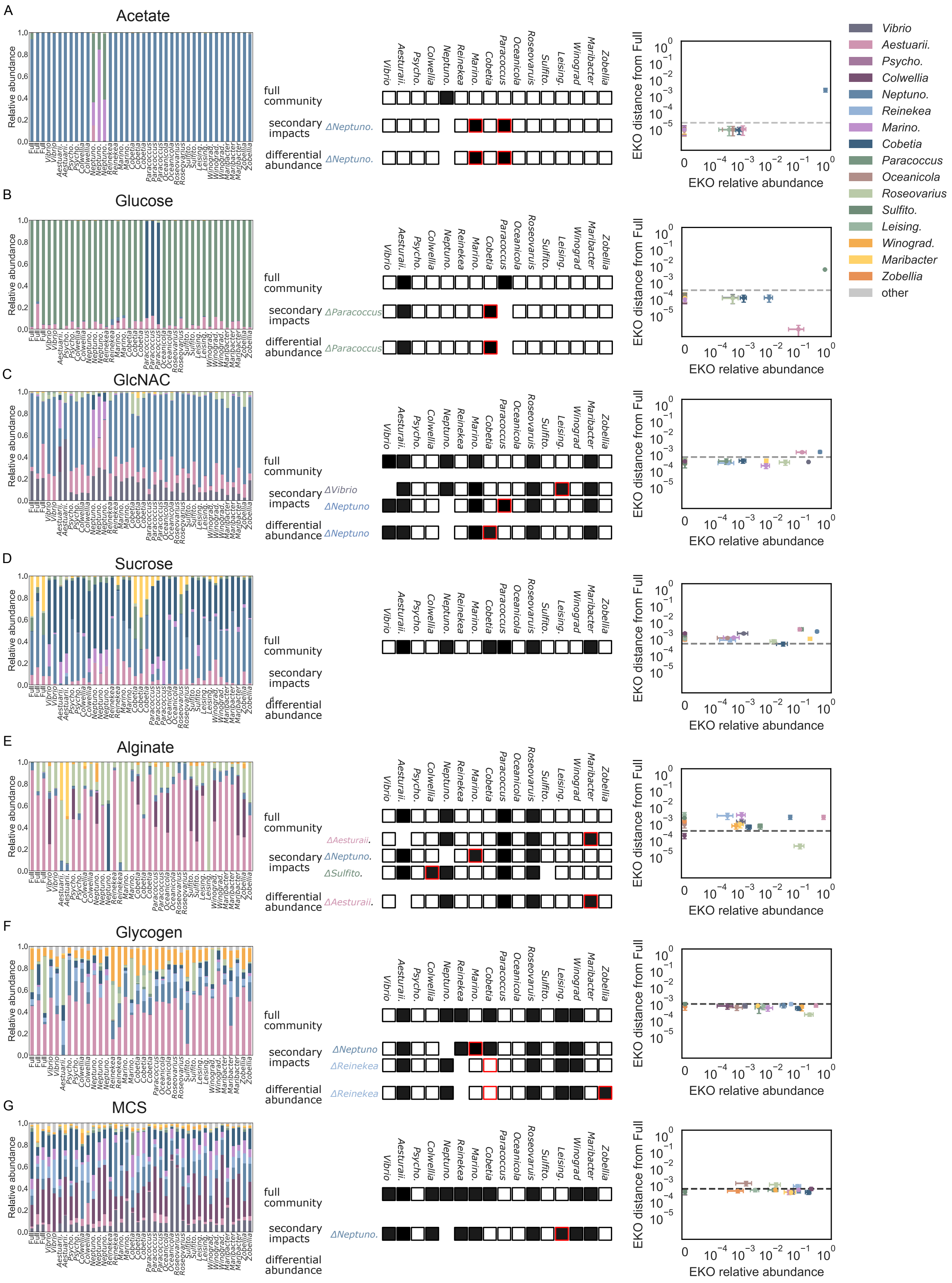

A
Acetate
B
Glucose
C
GlcNAC
D
Sucrose
E
Alginate
F
Glycogen
G
MCS
Relative abundance
full community
secondary impacts
differential abundance
ΔNeptuno.
ΔParacoccus
ΔVibrio
ΔAesturaii.
ΔSulfito.
ΔReinekea
Vibrio
Aesturaii.
Psycho.
Colwellia
Neptuno.
Reinekea
Marino.
Cobetia
Paracoccus
Oceanicola
Roseovarius
Sulfito.
Leising.
Winograd.
Maribacter
Zobellia
other
EKO distance from Full
EKO relative abundance

Supplementary Figure S1. Lack of keystone species is robust to the method used. Secondary impact analysis of all media (but raffinose which is presented in figure 2), as explained in Figure 2B-C. For each media we present the relative abundances of each community in each sample (left), a schematic illustration of species survival of the full(top) and all EKO communities in which an impact was identified either using secondary impacts (middle) or differential abundance (bottom). Each row represents a community, and each column represents a species. Filled and empty squares indicate survival and extinction, respectively. A red frame indicates a secondary impact where this species' presence differs from that in the full community (middle); The EKO distance from Full community of all EKOs. Circles are the mean of the computed distances between each EKO replicate and each prediction from full community replicate. The black dotted line is the mean+SE of all the differences between the three full community replicates (right).

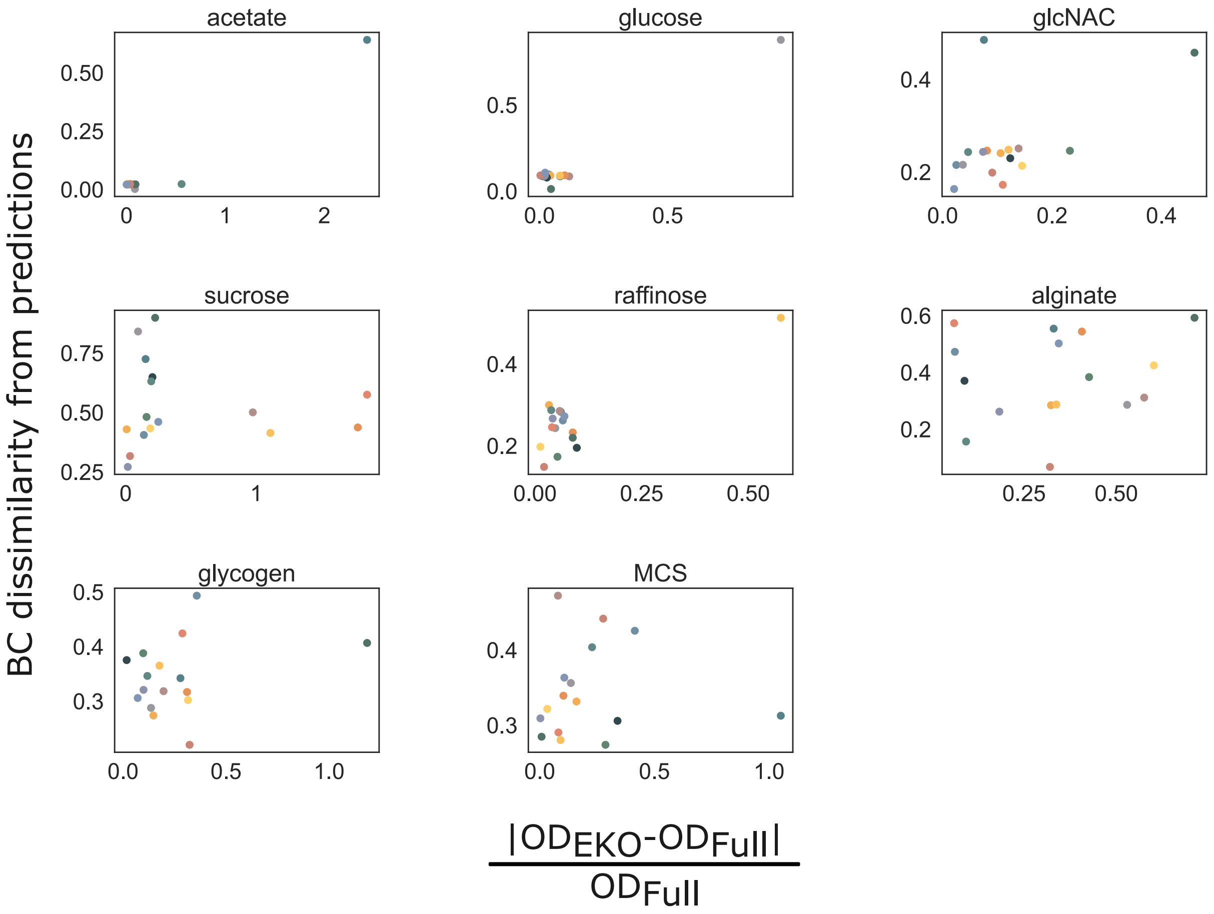

Supplementary Figure S2 Community shift vs. OD changes. For each EKO, its Bray-Curtis dissimilarity was calculated based on its prediction (normalized ASV frequencies after removing the EKO species reads from the full community). This was plotted against the normalized difference in OD of the EKO community compared to the full community. The two distance measures are correlated, with Spearman's rho = 0.6 and a p-value of 4.3×10^{-14}. Each dot represents the mean distances of all EKO replicates from the full community triplicates.

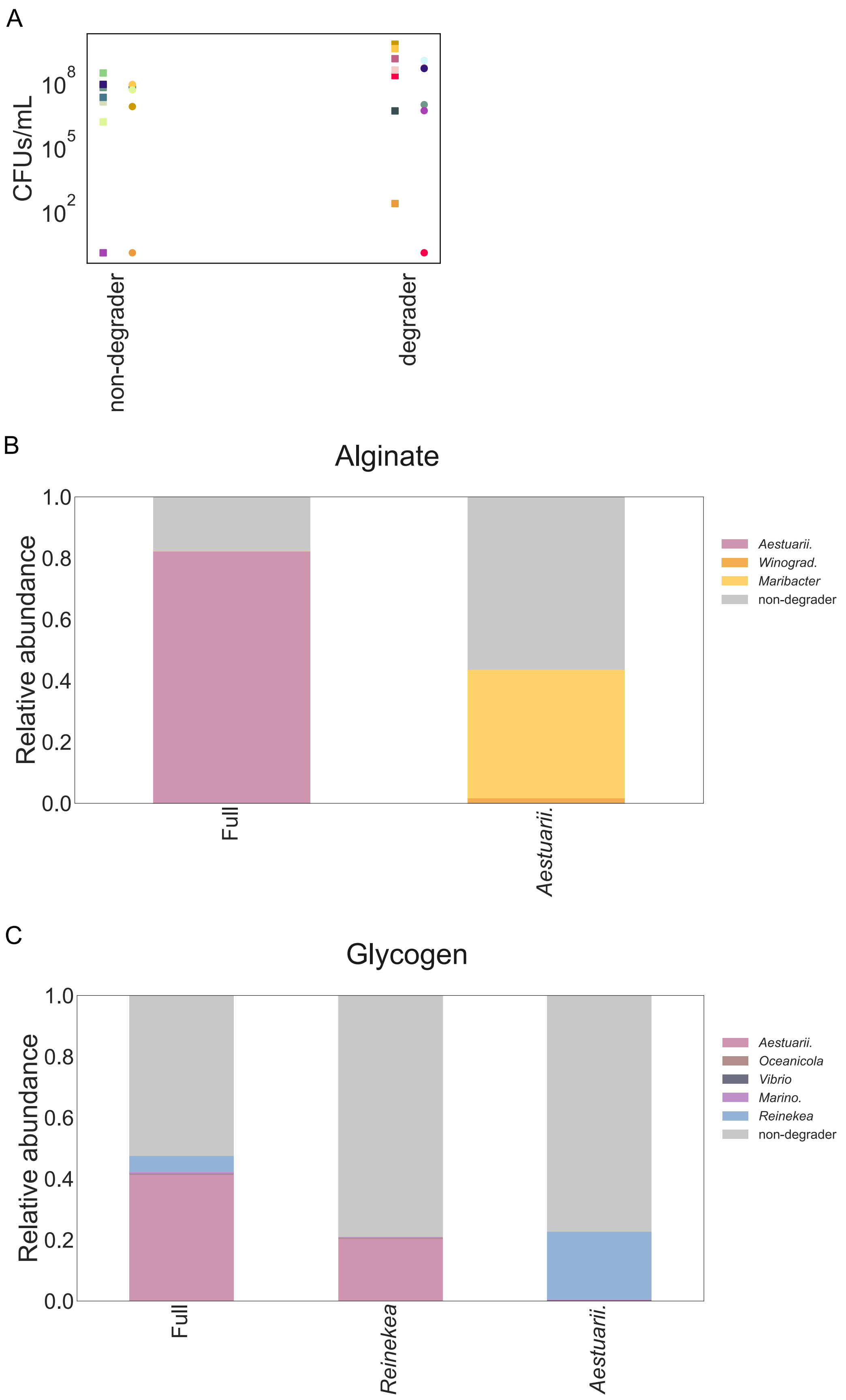
A
CFUs/mL
10^8
10^5
10^2
non-degrader
degrader
B
Alginate
Relative abundance
1.0
0.8
0.6
0.4
0.2
0.0
Full
Aestuarii.
Aestuarii.
Winograd.
Maribacter
non-degrader
C
Glycogen
Relative abundance
1.0
0.8
0.6
0.4
0.2
0.0
Full
Reinekea
Aestuarii.
Aestuarii.
Oceanicola
Vibrio
Marino.
Reinekea
non-degrader

Supplementary Figure S3. Metabolic ability to degrader the carbon source might underlie impacts following EKOs. A. capacities of all species as in figure 1, divided by having the ability to degrader either alginate (left columns) or glycogen (right columns). B-C. Mean relative abundance plots of the full and impacting degraders. Only species with a pathway to degrader alginate(B) or glycogen (C) appear distinctly, all others are in grey.

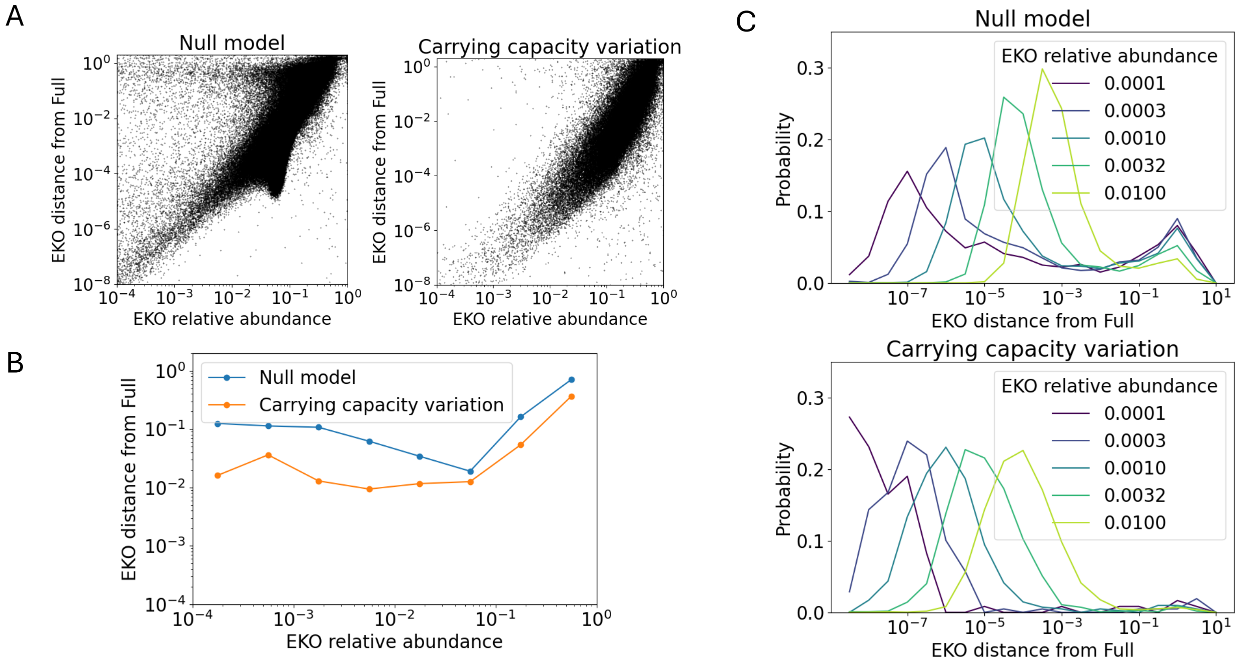

Supplementary Figure S4. Introduction of variation in carrying capacities reduces keystone species detected by EKO distance from Full. A. EKO distance form Full for EKO of species with a range of relative abundance. EKO distance from Full follows the definition in main text Fig. 2. EKOs with large relative abundance incur a large EKO distance from Full. However, under the Null model, many simulated communities exhibited significant EKO distances from Full even under EKO of species with small relative abundance. B. EKO distances from Full as a function of EKO relative abundance. Each point represents average over a window of EKO relative abundances. Over the whole range of EKO relative abundance, the Null model leads to larger EKO distances from Full than the Carrying capacity variation model does. C. Histogram of EKO distances from Full for small EKO relative abundances. The Null model exhibits a second peak at large EKO distances from Full, which represents the keystone species which exerts a disproportionately large impact on community assembly.

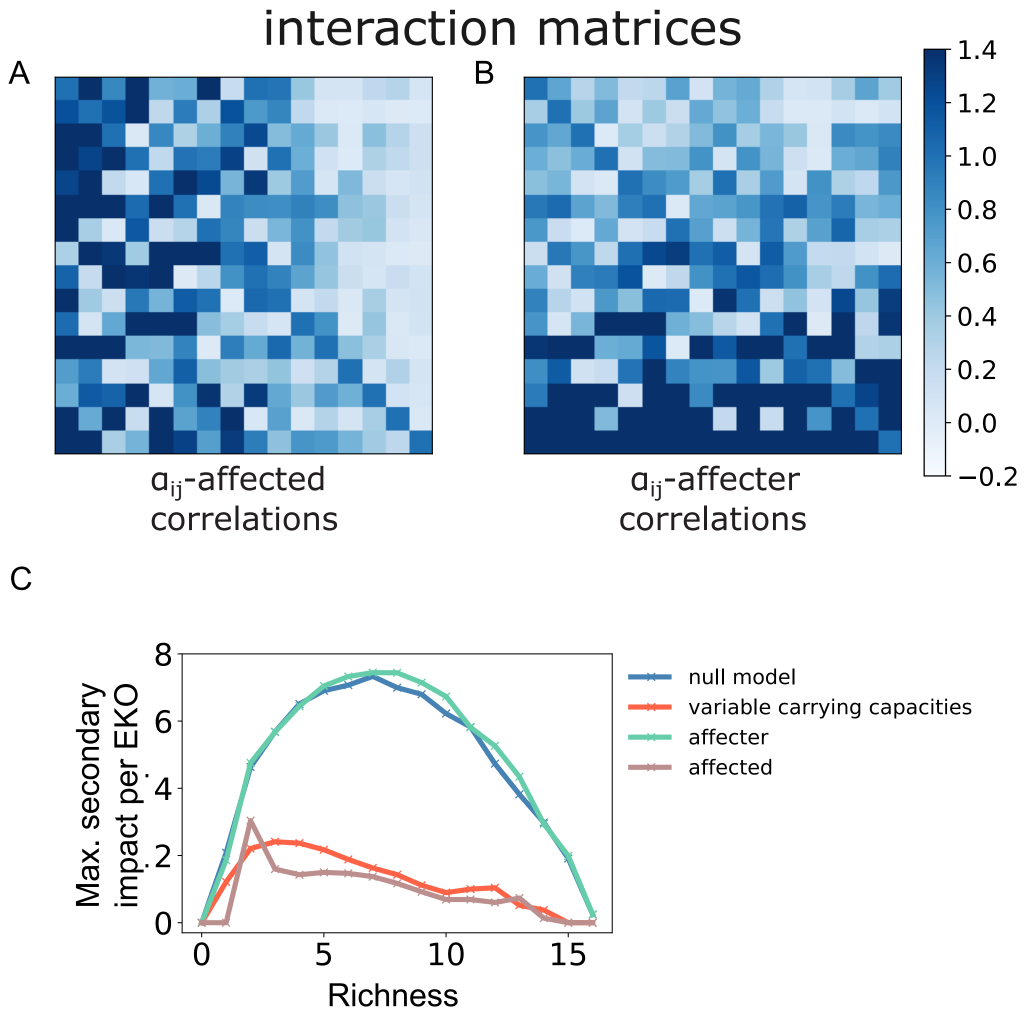

Supplementary Figure S5. Correlation of the affected interactions are the main contribution to the decrease in secondary impacts. **A-B.** Representative interaction matrices with correlations based on the affected species (A) and on the affecter species (B). The base interactions A_{ij} are drawn independently and identically distributed (i.i.d) from a certain distribution (a uniform distribution is shown here), and then the effects from carrying capacity $a_{ij} = \frac{K_j}{K_i} A_{ij}$ is applied only across different rows (A: $a_{ij} = \frac{1}{K_i} A_{ij}$) or across different columns (B: $a_{ij} = K_j A_{ij}$) of interaction matrices. **C**. Maximum secondary impacts per EKO as a function of the richness of the full community. 4 curves are shown: affected-based correlations, affecter-based correlations, carrying capacity-induced correlations, and no correlations (the null model). We find that both affected-based correlations and carrying capacity-induced correlations lead to decrease in secondary impacts, while affecter-based correlation does not change secondary impacts compared to the null model.

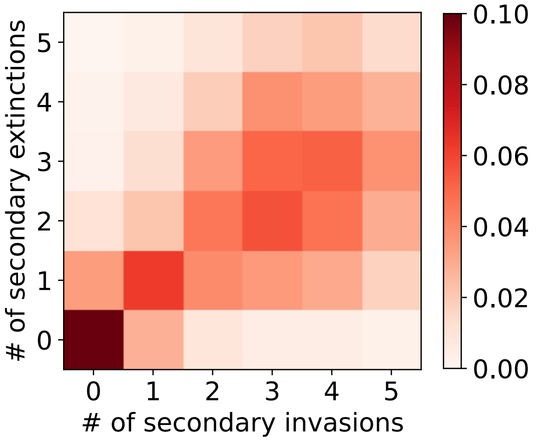

Supplementary Figure S6. Null model has a similar likelihood of secondary invasions and extinctions. Density plot of the frequency of null model simulations resulting in the stated number of secondary extinctions and invasions. The data is distributed along the line $x = y$, unlike in the case of carrying capacity simulations (Fig. 3C).

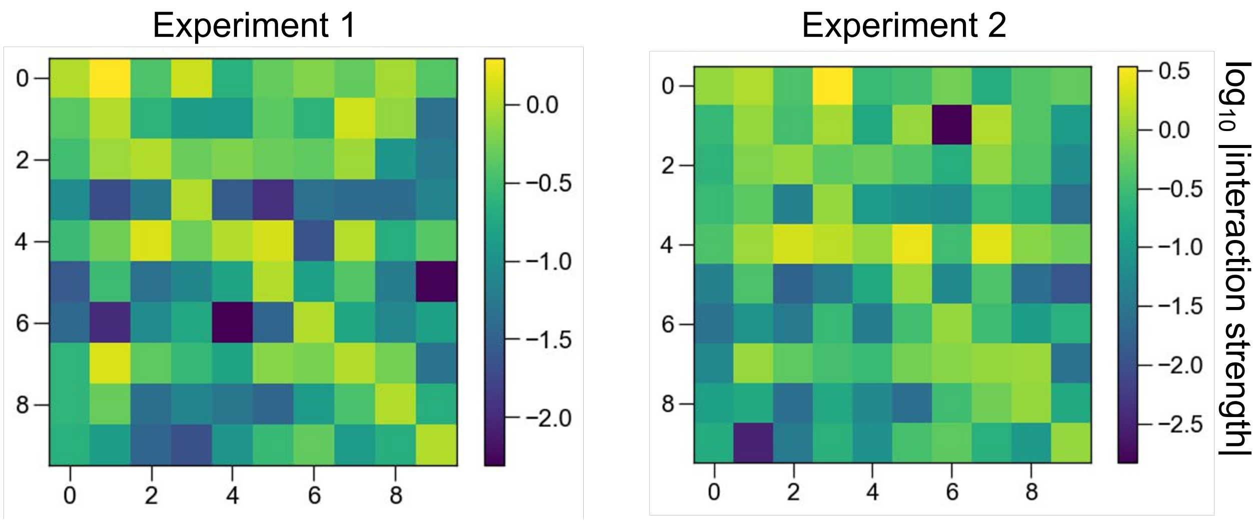

Supplementary Figure S7. Inferred interaction matrices for multiple environments. Interaction strength matrices for the 9 species that coexist in the glycogen communities inferred using data from two independent experimental replicates (left and right). The color bars represent the log of the magnitude of the interaction strength between pairs of species. The two matrices are strongly correlated (R = 0.88; $P < 10^{-3}$).

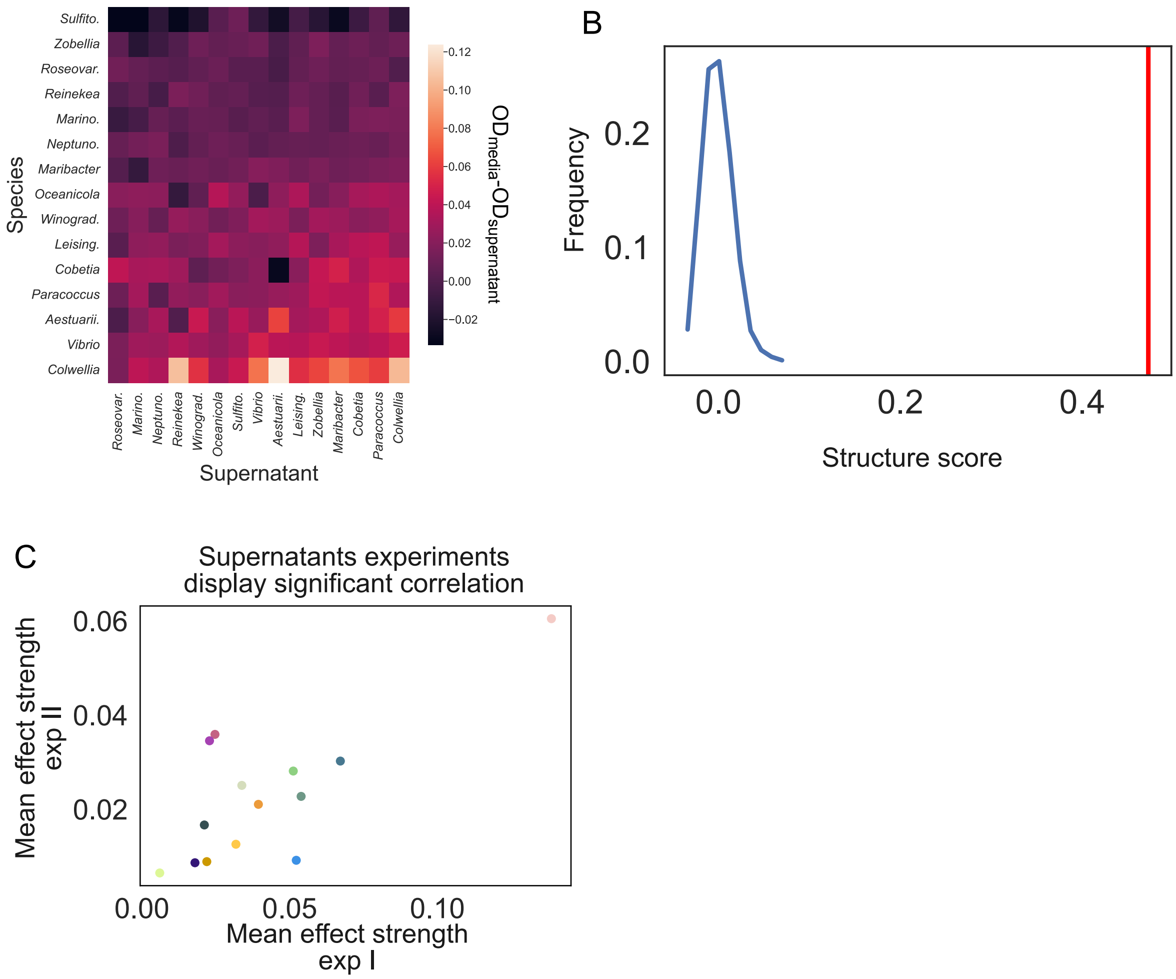

Supplementary Figure S8. Repetition of the spent media experiment show similar hierarchical structure. A. measured supernatants impacts. The rows are ordered by the least impacted species (mean impacts from all supernatants) to the most impacted. Similarly, columns are ordered by the least impacting supernatant to the most impacting. B. Hierarchical scores of the species correlations. In red the score of the measured matrix in A. In blue a histogram of the score of 1000 shuffles of the matrix entries. C. a scatter plot of the mean of each species impacts from the 2 experiments (the one from Figure 4 and this one). The Pearson correlation between them is significant (ρ= 0.755, p-value=0.0018).

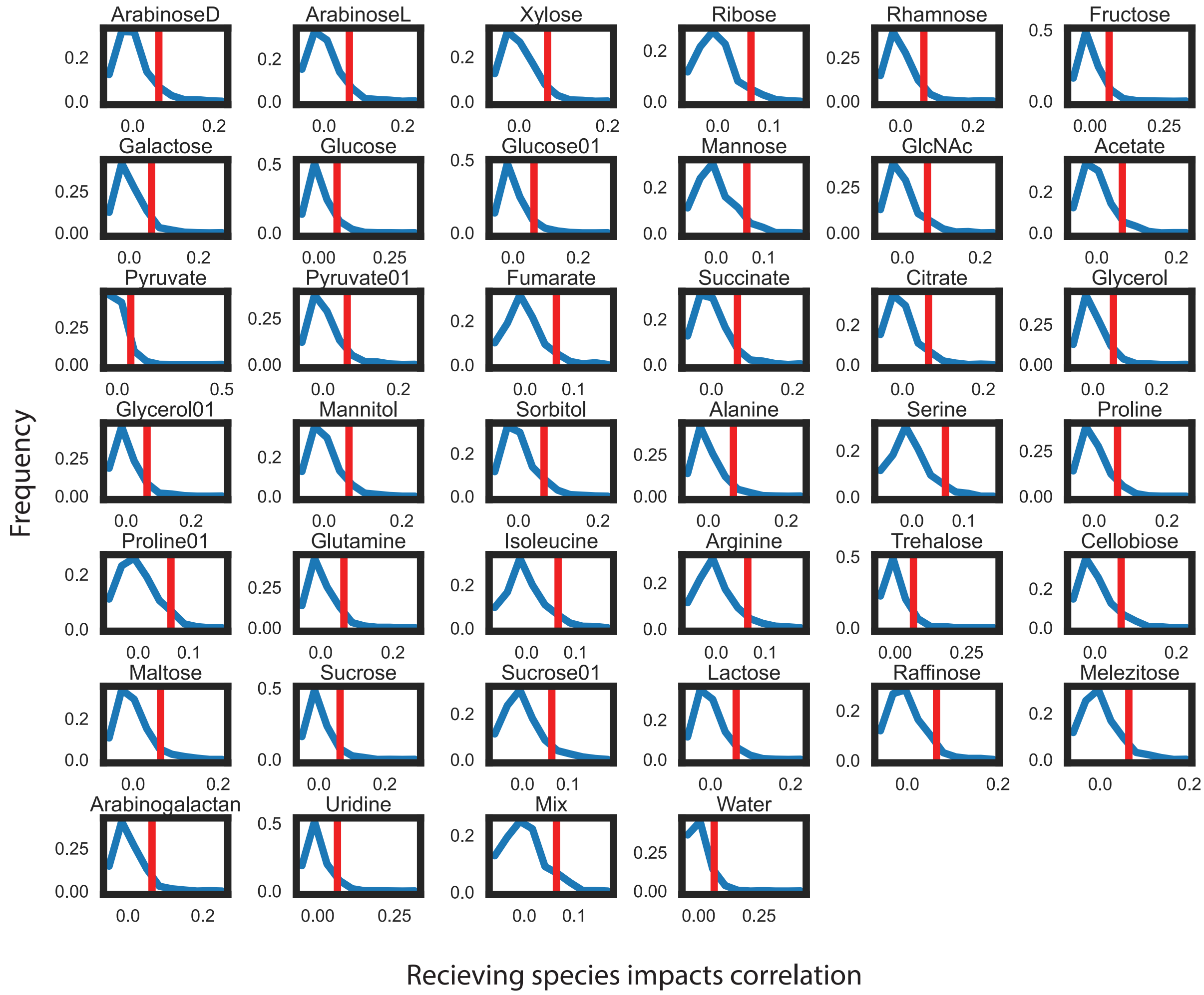

Supplementary Figure S9. Pairwise interactions measured using the K-chip display columns correlations. Structure scores quantifying the mean correlation between columns of an interaction matrix over 40 different carbon sources tested in the K-Chip[9]. In red- the score of the measured interaction matrix. In blue a histogram of the score of 1000 shuffles of the matrix entries.

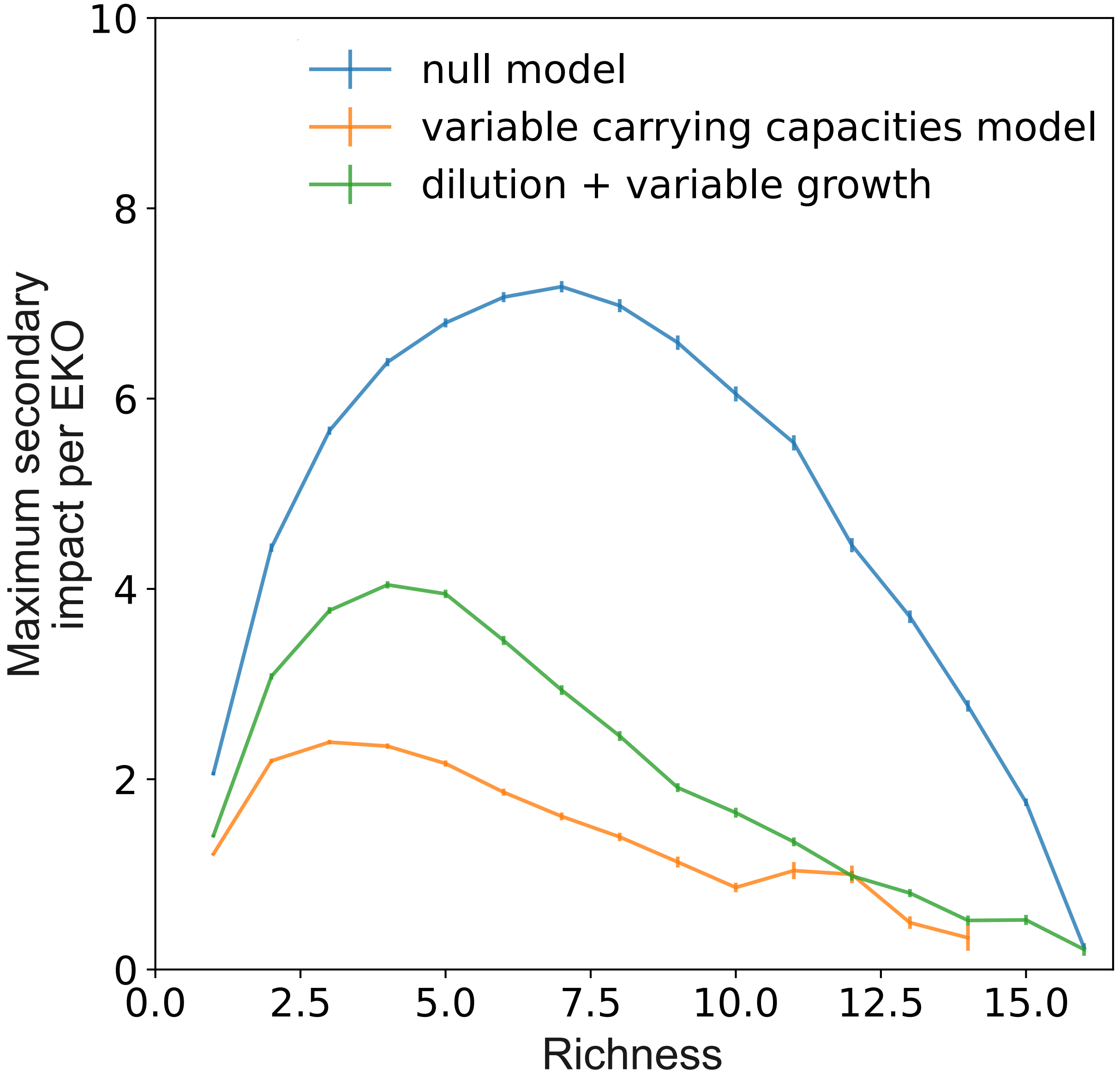

Supplementary Figure S10. Variable growth rate and dilution can reduce secondary impacts. Under dilution+variable growth condition, growth rates are evenly distributed across 16 species from 0.5 to 1.5 and a universal mortality of 0.4 is applied for all species. The plot shows results from 19000 communities as in the main text. We find that combining variation in growth rates and dilution can reduce the secondary impacts. This is because dilution rate structures the effective interaction matrix according to growth rate hierarchy[10].

Bibliography

1. Karp PD et al. The BioCyc collection of microbial genomes and metabolic pathways. *Brief Bioinform* 2019;**20**:1085–1093. https://doi.org/10.1093/bib/bbx085
2. Dal Bello M et al. Resource-diversity relationships in bacterial communities reflect the network structure of microbial metabolism. *Nat Ecol Evol* 2021;**5**:1424–1434. https://doi.org/10.1038/s41559-021-01535-8
3. Datta MS et al. Microbial interactions lead to rapid micro-scale successions on model marine particles. *Nat Commun* 2016;**7**:1–7. https://doi.org/10.1038/ncomms11965
4. Goldford JE et al. Emergent simplicity in microbial community assembly. *Science* 2018;**361**:469–474. https://doi.org/10.1126/science.aat1168
5. D'Souza G et al. Interspecies interactions determine growth dynamics of biopolymer-degrading populations in microbial communities. *Proc Natl Acad Sci U S A* 2023;**120**:e2305198120. https://doi.org/10.1073/pnas.2305198120
6. Ansari AF et al. An efficient and scalable top-down method for predicting structures of microbial communities. *Nat Comput Sci* 2021;**1**:619–628. https://doi.org/10.1038/s43588-021-00131-x
7. Xiao Y et al. Mapping the ecological networks of microbial communities. *Nat Commun* 2017;**8**:2042. https://doi.org/10.1038/s41467-017-02090-2
8. McLaren MR, Willis AD, Callahan BJ. Consistent and correctable bias in metagenomic sequencing experiments. *eLife* 2019;**8**:e46923. https://doi.org/10.7554/eLife.46923
9. Kehe J et al. Positive interactions are common among culturable bacteria. *Sci Adv* 2021;**7**:eabi7159. https://doi.org/10.1126/sciadv.abi7159

10. Abreu CI et al. Mortality causes universal changes in microbial community composition. *Nat Commun* 2019;**10**:2120. https://doi.org/10.1038/s41467-019-09925-0
